# Supplementary material for: Cloud BioLinux: pre-configured and on-demand bioinformatics computing for the genomics community
Source: BMC Bioinformatics. 2012 Mar 19;13:42. doi: 10.1186/1471-2105-13-42 (PMC3372431; doi:10.1186/1471-2105-13-42)
Supplement: Additional file 1 — Supplementary 1 Cloud BioLinux software documentation in the form of a mini, self-contained website. Users need to download and uncompress the .zip file, and open through a web browser the "index.html" file available on the main directory. (ZIP 1823 kb). [file 1471-2105-13-42-S1.ZIP › Cloud-BioLinux-Package-Documentation/docs/treedist.html]

Bio-Linux Software Documentation Pages

Back to search form

## treedist

|  |  |
| --- | --- |
| Name | treedist |
| Description | **treedist** is part of the PHYLIP package  � Copyright 2000-2004 by The University of Washington. Written by Joseph Felsenstein. Permission is granted to copy this document provided that no fee is charged for it and that this copyright notice is not removed.  This program computes distances between trees. Two distances are computed, the Branch Score Distance of Kuhner and Felsenstein (1994), and the more widely known Symmetric Difference of Robinson and Foulds (1981). The Branch Score Distance uses branch lengths, and can only be calculated when the trees have lengths on all branches. The Symmetric Difference does not use branch length information, only the tree topologies. It must also be borne in mind that neither distance has any immediate statistical interpretation -- we cannot say whether a larger distance is significantly larger than a smaller one. |
| Homepage | http://evolution.genetics.washington.edu/phylip.html |
| Remote Documentation | http://evolution.genetics.washington.edu/phylip/doc/treedist.html |
